# Thoracic Dorsal Root Ganglion Application of Resiniferatoxin Reduces Myocardial Ischemia-Induced Ventricular Arrhythmias

**DOI:** 10.3390/biomedicines11102720

**Published:** 2023-10-07

**Authors:** Tomoki Yamaguchi, Siamak Salavatian, Yuki Kuwabara, Abigail Hellman, Bradley K. Taylor, Kimberly Howard-Quijano, Aman Mahajan

**Affiliations:** 1 Department of Anesthesiology and Perioperative Medicine, University of Pittsburgh, Pittsburgh, PA 15261, USA; tomo-yamaguchi2000@hotmail.co.jp (T.Y.); salavatian@pitt.edu (S.S.); yukik@pitt.edu (Y.K.); ash148@pitt.edu (A.H.); bkt@pitt.edu (B.K.T.); howardquijanokj@upmc.edu (K.H.-Q.); 2 Division of Cardiology, Department of Medicine, University of Pittsburgh Medical Center, Pittsburgh, PA 15261, USA; 3 Department of Anesthesiology and Perioperative Medicine, University of Pittsburgh Medical Center, Pittsburgh, PA 15261, USA

**Keywords:** neuromodulation, myocardial ischemia, cardiac arrhythmias, deafferentation, autonomic nervous system, RTX

## Abstract

Background: A myocardial ischemia/reperfusion (IR) injury activates the transient receptor potential vanilloid 1 (TRPV1) dorsal root ganglion (DRG) neurons. The activation of TRPV1 DRG neurons triggers the spinal dorsal horn and the sympathetic preganglionic neurons in the spinal intermediolateral column, which results in sympathoexcitation. In this study, we hypothesize that the selective epidural administration of resiniferatoxin (RTX) to DRGs may provide cardioprotection against ventricular arrhythmias by inhibiting afferent neurotransmission during IR injury. Methods: Yorkshire pigs (*n* = 21) were assigned to either the sham, IR, or IR + RTX group. A laminectomy and sternotomy were performed on the anesthetized animals to expose the left T2–T4 spinal dorsal root and the heart for IR intervention, respectively. RTX (50 μg) was administered to the DRGs in the IR + RTX group. The activation recovery interval (ARI) was measured as a surrogate for the action potential duration (APD). Arrhythmia risk was investigated by assessing the dispersion of repolarization (DOR), a marker of arrhythmogenicity, and measuring the arrhythmia score and the number of non-sustained ventricular tachycardias (VTs). TRPV1 and calcitonin gene-related peptide (CGRP) expressions in DRGs and CGRP expression in the spinal cord were assessed using immunohistochemistry. Results: The RTX mitigated IR-induced ARI shortening (−105 ms ± 13 ms in IR vs. −65 ms ± 11 ms in IR + RTX, *p* = 0.028) and DOR augmentation (7093 ms^2^ ± 701 ms^2^ in IR vs. 3788 ms^2^ ± 1161 ms^2^ in IR + RTX, *p* = 0.020). The arrhythmia score and VT episodes during an IR were decreased by RTX (arrhythmia score: 8.01 ± 1.44 in IR vs. 3.70 ± 0.81 in IR + RTX, *p* = 0.037. number of VT episodes: 12.00 ± 3.29 in IR vs. 0.57 ± 0.3 in IR + RTX, *p* = 0.002). The CGRP expression in the DRGs and spinal cord was decreased by RTX (DRGs: 6.8% ± 1.3% in IR vs. 0.6% ± 0.2% in IR + RTX, *p* < 0.001. Spinal cord: 12.0% ± 2.6% in IR vs. 4.5% ± 0.8% in IR + RTX, *p* = 0.047). Conclusions: The administration of RTX locally to thoracic DRGs reduces ventricular arrhythmia in a porcine model of IR, likely by inhibiting spinal afferent hyperactivity in the cardio–spinal sympathetic pathways.

## 1. Introduction

Sudden cardiac death may occur as a result of ventricular arrhythmia induced by myocardial ischemia [1]. Although pharmacologic treatment and catheter ablation therapies are available, they often provide inadequate control of arrhythmia [2]. Myocardial ischemia causes the excitation of cardiac afferent nerves, including myelinated A-delta and unmyelinated C fibers expressing transient receptor potential vanilloid 1 (TRPV1), which are associated with arrhythmogenic ventricular remodeling [3,4]. To improve the management of ventricular arrhythmias, novel therapies such as neuromodulation have garnered significant interest [5]. Neuromodulation therapies target the cardiac autonomic nervous system and provide cardioprotection by decreasing the sympathetic tone or increasing the parasympathetic tone through various methods [5].

RTX is a selective ultra-potent TRPV1 agonist, and it has been shown that it can induce a prolonged augmentation in intracellular calcium in vanilloid-sensitive neurons in human dorsal root ganglion (DRG) cultures while not affecting the adjacent neurons [6,7,8,9,10]. RTX is a toxic activator of TRPV1 that can lead to the desensitization of the receptor [6,8,11,12]. RTX has been administered pericardially as a therapeutic approach to inhibit the local afferent neurons in the heart and reduce ventricular arrhythmogenicity [13]. However, this method is invasive and can increase blood pressure, making it unsuitable for some cardiac patients [4]. Recent studies suggest that the intrathecal administration of RTX can effectively suppress ventricular arrhythmia [14,15]. Further, studies in rats indicate that the epidural administration of RTX can prevent hypertension by suppressing the cardiac afferent reflex [16].

In this study, we evaluated the efficacy of a selective local application of RTX to the DRG in mitigating ventricular arrhythmogenicity in a preclinical model. To gain a better understanding of the underlying mechanism, we employed immunohistochemistry techniques.

## 2. Methods

Yorkshire pigs (49 kg ± 1 kg, 10 males, 11 females) were used in this study. The protocol was approved by the Institutional Animal Care and Use Committee (Code: 18103849, approval date: 24 October 2018, University of Pittsburgh, Pittsburgh, PA, USA). The National Institution of Health Guide for the Care and Use of Laboratory Animals was followed when conducting the experiments. Animals were euthanized by the intravenous administration of potassium chloride (100 mg/kg, 30 mg/mL) under general anesthesia (Isoflurane, 5% inh.).

### 2.1. Surgical Preparation

Intramuscular Telazol (4 mg/kg, 100 mg/mL) and xylazine (2 mg/kg, 100 mg/mL) were used to sedate the animal, following isoflurane inhalation at 1% to 2% for general anesthesia. The animals were intubated, and mechanical ventilation was initiated. Electrocardiogram (ECG), oxygen saturation, blood pressure, and body temperature were monitored. Catheters were also inserted into both the left and right external jugular veins to administer fluids and medications. A laminectomy was initiated while the animal was in the prone position and the T2–T4 dorsal roots were exposed. The animal was returned to the supine position, and a sternotomy was performed to expose the heart. An epicardial sock electrode array was placed over the heart to acquire electrophysiological data. Data recording was performed in the left lateral position to allow the administration of RTX into the left DRGs through the epidural space. As previously described, during data acquisition, general anesthesia was switched to alpha chloralose (50 mg/kg initial bolus followed by a 20 mg/kg/h continuous infusion, 50 mg/mL) to minimize the effect of anesthesia on autonomic function [17]. ECG data were continuously acquired and recorded on the Prucka CardioLab System (GE Healthcare, Chicago, IL, USA). Body temperature was maintained using a warming blanket (Bair Hugger Underbody Warming Blankets, 3 M, Dubai, United Arab Emirates). An arterial blood gas analysis was performed, and the mechanical ventilation settings were adjusted as needed.

### 2.2. Acute Myocardial Ischemia and Reperfusion

A previously validated method of creating the acute myocardial ischemia model was used in this study [18]. A Prolene suture was placed around the left anterior descending coronary artery (LAD) below the second diagonal branch. A short polyethylene tube was passed through both ends of the suture, and blood flow was disrupted by pulling the suture and fixing pean forceps from the distal end of the tube. Acute myocardial ischemia was performed for 1 h, after which the suture was released, followed by 2 h of reperfusion. The ST elevation on the ventricular epicardial electrogram was used to confirm myocardial ischemia. In the event of pulseless ventricular tachycardia or ventricular fibrillation, we followed advanced cardiovascular life support guidelines and resuscitated the animal with internal cardiac defibrillation, cardiac massage, and epinephrine [19]. If the animal did not return to spontaneous circulation despite resuscitation, the suture was released and resuscitation was continued. The reperfusion was monitored for a period of two hours following the pigs’ restoration of spontaneous circulation.

### 2.3. Epidural Administration of RTX to DRGs

**Figure 1:**
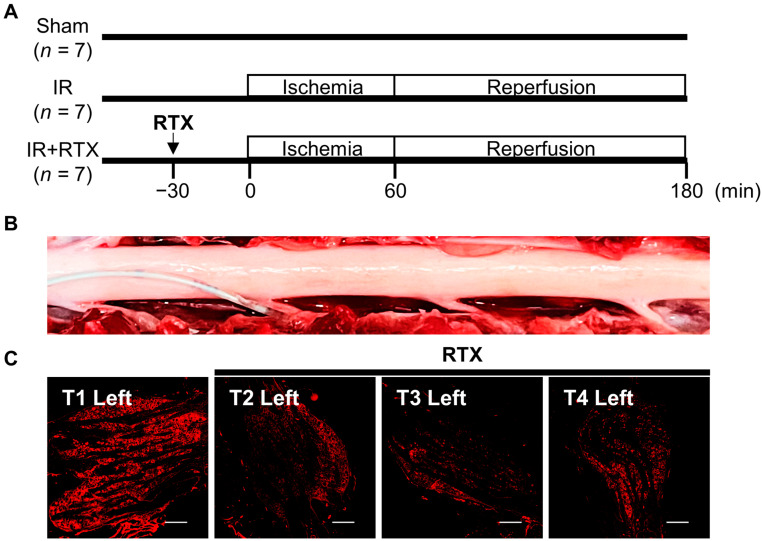
The study protocol and application of RTX to thoracic DRG. (**A**) Yorkshire pigs were assigned to 3 groups, sham, IR, and IR + RTX. The vehicle was not administered to animals in the sham and IR groups. (**B**) The panel shows the dorsal surface of the T1–T4 spinal cord, with the left nerve root at the bottom. The catheter is inserted into the left T2 dorsal root. RTX was administered to the left T2, T3, and T4 DRGs sequentially. (**C**) Immunofluorescent images of TRPV1 in the left DRGs. T2–T4 injected RTX showed reduced expression of TRPV1 compared to T1. Scale bar = 500 μm. IR = ischemia/reperfusion; IR + RTX = ischemia/reperfusion + resiniferatoxin; T1 = thoracic spinal nerve 1; TRPV1 = transient receptor potential vanilloid 1; DRG = dorsal root ganglion.

The animal was placed in the left lateral position and the left dorsal root of the spinal cord was identified after surgical preparation. The epidural catheter was advanced along the left dorsal root of the T2 level. The catheter tip was placed in the epidural space just above the DRG (Figure 1). RTX (100 μg/mL) (AdipoGen Life Sciences, San Diego, CA, USA) was administered over 1 min in 0.5 mL doses to the left T2, T3, and T4 DRGs sequentially. After the administration of RTX to each level, the catheter was removed. RTX was prepared according to the composition previously reported [16]. RTX was dissolved in a 1:1:8 mixture of dimethyl sulfoxide (Sigma-Aldrich, St. Louis, MO, USA), Tween 80 (Fisher Scientific, Waltham, MA, USA) and isotonic saline.

### 2.4. Experimental Protocols

Twenty-one animals were used, and they were randomly assigned into three groups, as shown in Figure 1. The vehicle was not administered to animals in the sham and ischemia/reperfusion (IR) groups.

### 2.5. Hemodynamic Recording

To evaluate the hemodynamic changes caused by RTX administration, heart rate was measured by a lead II ECG, and systolic, mean, and diastolic blood pressures were obtained from a femoral arterial blood pressure monitor using the Prucka Cardiolab system (GE Healthcare, Chicago, IL, USA).

To measure left ventricular pressure, a pressure catheter (5 French SPR350, Millar Instruments, Houston, TX, USA) was inserted through the left internal carotid artery into the left ventricle and connected to the MPVS Ultra Pressure–Volume Loop System (Millar Instruments, Houston, TX, USA). The left ventricular end-systolic pressure (LVESP), the maximum rate of rise in left ventricular pressure (dP/dt max), and the minimum rate of left ventricular pressure change (dP/dt min) were collected to evaluate left ventricular function.

### 2.6. Heart Staining

After the completion of the entire procedure, the LAD was ligated and a cross-clamp was used on the ascending aorta and pulmonary veins and 2% Evans Blue dye (MP Biomedicals, Irvine, CA, USA) was injected below the cross-clamp in the aorta with a butterfly needle, and then the heart was pumped manually to distribute the dye throughout the heart. The area not stained by Evans blue dye on the heart slices was identified as the area at risk (%).

### 2.7. Cardiac Electrophysiologic Recording

To measure unipolar electrograms, a 56-electrode sock array was placed around the epicardium of the heart and connected to a Prucka CardioLab electrophysiology mapping system (GE Healthcare, Chicago, IL, USA). This electrode array allows continuous in vivo recording of local electrograms throughout the whole heart during the entire experiment. The obtained electrogram was used to calculate the activation recovery interval (ARI), which is a surrogate for action potential duration in the myocardium [18,20,21]. The ARI calculation was performed using customized software (iScalDyn; University of Utah) [18,20,21]. The ARI was used to assess the myocardial APD. The ARI data were analyzed for the global heart and in ischemic and non-ischemic regions defined by ST-segment changes, following the previously described guidelines [22]. The dispersion of repolarization (DOR) was calculated using the variance of all the acquired ARIs. An increasing heterogeneity of repolarization throughout the heart is associated with the incidence of ventricular arrhythmia [21].

### 2.8. Arrhythmia Counts

An arrhythmia assessment was performed during 1 h of ischemia and 1 h of reperfusion. We manually counted the number of premature ventricular contractions (PVCs), non-sustained ventricular tachycardia (VT), and ventricular fibrillation (VF) using the Prucka CardioLab system. PVCs were defined by a premature QRS complex, and VT was identified by 3 or more consecutive PVCs [23]. The arrhythmia score was calculated as (log_10_ PVCs) + (log_10_ episodes VT) + 2 [(log_10_ episodes of VF) + (log_10_ total duration of VF)] and was used to assess the ventricular arrhythmogenicity [24].

### 2.9. Immunohistochemistry (IHC)

After the animals were euthanized, neural tissue, including T1–T4 spinal cord segments and DRGs, were collected. Tissues were rinsed in cold 0.1 M phosphate-buffered saline (PBS) to remove blood and placed into 4% paraformaldehyde (Thermo Scientific, Waltham, MA, USA) for 24 h at 4 °C following dissection. Then, samples were placed into 30% sucrose with PBS containing 0.01% sodium azide at 4 °C until the tissues sank. Fixed tissues were embedded in an optimum cutting temperature compound (Fisher Scientific, Waltham, MA, USA) and stored at −80 °C until the experiments were completed. The spinal cord and DRG tissue were sectioned using a Cryostat at 35 μm, and 10 μm, respectively (Cryostar NX50; Thermo Fisher Scientific, Waltham, MA, USA). DRG staining was performed on-slide, while spinal cord staining was performed free-floating in well plates.

To perform IHC, sections were blocked in PBS with 5% normal donkey serum (Jackson Immuno Research Laboratories Inc., Cambridgeshire, UK) for 1 h at room temperature. They were then incubated in a blocking solution and 0.3% Triton x-100 (Sigma-Aldrich, St. Louis, MO, USA) with primary antibodies overnight at 4 °C (16–22 h). The primary antibodies used include guinea pig anti-TRPV1 (1:100; Neuromics, Edina, MN, USA, cat. no. GP14100), mouse anti-CGRP (1:500; Abcam, Cambridge, UK, cat. no. Ab81887), and rabbit anti-NeuN (1:500; Abcam, cat. no. Ab177487). After primary incubation, the tissue was washed in PBS three times at 5 min intervals after which the tissue was incubated in a blocking solution with a secondary antibody for 1 h at room temperature. The secondary antibodies included Cy3 conjugated donkey anti-guinea pig (1:500; Millipore Sigma, Burlington, MA, USA, cat. no. AP193 C), Cy3 Affinipure donkey anti-mouse (1:750; Jackson ImmunoResearch Laboratories Inc., Chester County, PA, USA, cat. no. 715-165-150), Alexa fluor donkey anti-rabbit 488 (1:500; Invitrogen, Waltham, MA, USA cat. no. A-21206), and Alexa fluor donkey anti-mouse 647 (1:500; Invitrogen, Waltham, MA, USA cat. no. A-31571). After their incubation in the secondary antibody, sections were washed in PBS again (3 × 5 min), and then spinal tissue was mounted onto microscope slides (Superfrost, Fisher Scientific, Waltham, MA, USA). All slides were coverslipped with Vectarshield Hardset Antifade Mounting Medium with DAPI (H-1500, Vector Laboratories, Newark, CA, USA).

Imaging was performed with a Nikon Eclipse Ti2 Inverted Microscope System using NIS-Elements AR Imaging Software V 5.10.01 (Nikon Instruments Inc., Melville, NY, USA). Images were taken at 20× or 40×.

Images were analyzed by a blinded investigator and analysis protocols were standardized to avoid experimental bias. The mean from two slices per animal was analyzed per spinal segment. The region of interest (ROI) for DRG neurons was set based on NeuN, a neuronal marker, and the ROI for the spinal cord was based on the bilateral dorsal horn, where the afferent nerve endings from the DRG project to dorsal horn laminae I and II [10]. All tissues were analyzed based on the mean intensity of consistent thresholds across the target protein. Data were averaged and analyzed by group, left vs. right side for the IR + RTX group.

### 2.10. Statistical Analysis

The sample size was determined based on our previous publication in which a similar experimental protocol was performed and electrophysiologic recordings during myocardial ischemia were compared [18]. In advance, the Shapiro–Wilk test or Kolmogorov–Smirnov test was used to check if the data followed a normal distribution. For parametric data sets, a two-tailed student’s *t*-test and a one-way ANOVA test were performed. For non-parametric data sets, a Mann–Whitney test and Kruskal–Wallis test were performed. If significant changes were reported by a one-way ANOVA test or Kruskal–Wallis test, this was followed by a post hoc multiple comparisons test. For the VF occurrence, the Fisher’s exact test was used to compare the IR and IR + RTX groups. A two-tailed paired student’s *t*-test for parametric data sets and a Wilcoxon matched-pairs signed rank test for non-parametric data sets were used to compare paired values between the same individuals. All continuous variables are presented as mean ± standard error of the mean (SEM). For all statistical tests, a *p* value < 0.05 was considered statistically significant. The analysis was performed using GraphPad Prism V 9.3.0 (GraphPad Software, La Jolla, CA, USA).

## 3. Results

### 3.1. Ischemia Area at Risk Assessment

To ensure that the myocardial ischemia episodes are comparable in the LAD vs. LAD+RTX group, the area at risk was assessed in the IR and IR + RTX groups. The ischemic regions were not different in these two groups (IR: 23% ± 3%, *n* = 7 vs. IR + RTX: 24% ± 2%, *n* = 7, *p* = 0.918).

### 3.2. Hemodynamic Changes

To evaluate the hemodynamic changes induced by the application of RTX to the unilateral thoracic DRG, hemodynamic parameters were recorded at baseline and after 5 and 30 min of RTX administration in the IR + RTX group. There was no significant change in hemodynamic parameters after the RTX administration compared to the baseline parameters (Table 1).

To evaluate the hemodynamic changes during myocardial ischemia, the hemodynamic parameters were recorded at baseline and 30 min after the onset of ischemia (or right before VT in case the animal experienced VT during ischemia) (Table 2). In the sham group, hemodynamic parameters were recorded at baseline and at 30 min without ischemia. The IR decreased the LVESP (baseline: 117 ± 8 mmHg to LAD30: 111 ± 9 mmHg, *p* = 0.025) and LVdp/dt max (baseline: 2117 ± 186 mmHg/s to LAD30: 1804 ± 167 mmHg/s, *p* = 0.022). The LVESP (baseline: 114 ± 8 mmHg to LAD30: 110 ± 7 mmHg, *p* = 0.51) and LVdp/dt max (baseline: 1826 ± 127 mmHg/s to LAD30: 1677 ± 75 mmHg/s, *p* = 0.11) were not reduced significantly during the ischemia in the IR + RTX group. No other significant changes in the hemodynamic parameters were observed.

### 3.3. Cardiac Electrophysiological Changes

**Figure 2:**
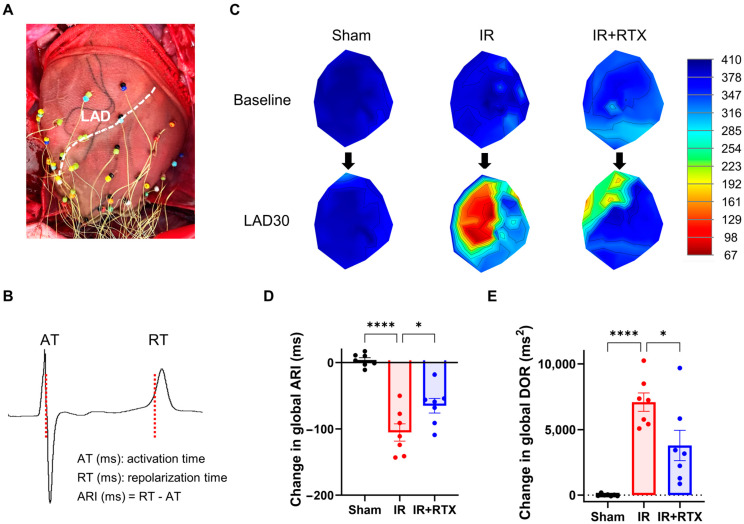
Effect of RTX on electrophysiological changes at 30 min after the onset of ischemia. (**A**) To measure unipolar electrograms, a 56-electrode sock mesh array was put around the epicardium of the heart. (**B**) The obtained electrogram was used to calculate the activation recovery interval (ARI), which is a surrogate measurement for action potential duration in the myocardium. (**C**) The representative polar maps demonstrate the change in ARI from baseline to 30 min after the onset of ischemia. (**D**,**E**) The change in global ARI and DOR at 30 min after the onset of ischemia was compared between the groups. The application of RTX attenuated the change in global ARI and DOR. sham: *n* = 7, IR: *n* = 5, IR + RTX: *n* = 7; data are shown as mean ± SEM. * *p* < 0.05, **** *p* < 0.0001 by one-way ANOVA test, followed by post hoc multiple comparisons test. LAD = left anterior descending coronary artery; LAD30 = 30 min after the onset of ischemia; ARI = activation recovery interval. DOR = dispersion of repolarization.

To evaluate myocardial ischemia-induced sympathetic activation, the ARI changes from baseline to during ischemia (30 min after the ischemia onset or right before VT in case the animal experienced VT during ischemia) were compared in the IR and IR + RTX groups for global, ischemic, and non-ischemic regions. The reduction in the global ARI (sham: 4 ms ± 4 ms, *n* = 7, IR: −105 ms ± 13 ms, *n* = 7, IR + RTX: −65 ms ± 11 ms, *n* = 7; IR vs. IR + RTX, *p* = 0.028) and the ischemic region ARI (IR: −181 ms ± 19 ms, *n* = 7, IR + RTX: −129 ms ± 12 ms, *n* = 7; IR vs. RTX, *p* = 0.030) due to ischemia was mitigated in the IR + RTX group compared to the IR group (Figure 2). In the non-ischemic region, the ARI shortened during the ischemia but not as much as in the ischemic region, and this ARI reduction was mitigated by the RTX (IR: −54 ms ± 11 ms, *n* = 7, IR + RTX: −20 ms ± 7 ms, *n* = 7; IR vs. IR + RTX, *p* = 0.026).

The increase in the DOR at 30 min after the onset of ischemia was significantly attenuated in the IR + RTX group compared to the IR group (sham: 7 ms^2^ ± 34 ms^2^, *n* = 7, IR: 7093 ms^2^ ± 701 ms^2^, *n* = 7, IR + RTX: 3788 ms^2^ ± 1161 ms^2^, *n* = 7; IR vs. IR + RTX, *p* = 0.020) (Figure 2).

### 3.4. Ventricular Arrhythmia

**Figure 3:**
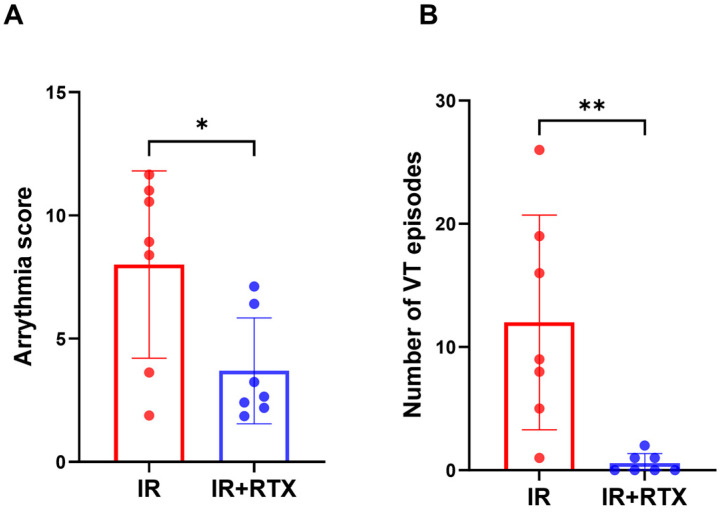
Effect of RTX on ventricular arrhythmia during ischemia. (**A**) The arrhythmia score during IR was decreased in the IR + RTX group compared to the IR group. For this score, the equation is as follows: (log_10_ PVCs) + (log_10_ episodes VT) + 2 [(log_10_ episodes of VF) + (log_10_ total duration of VF)]. (**B**) The number of VTs during IR was reduced in the IR + RTX group compared to the IR group. *n* = 7 per group; data are shown as mean ± SEM. * *p* < 0.05, ** *p* < 0.01, by one-way ANOVA test for the arrhythmia score and Kruskal-Wallis test for Number of VT, followed by post hoc multiple comparisons test.

The arrhythmia score and the number of VTs in each group were calculated to determine the arrhythmia risk between the groups. During the IR injury, the arrhythmia score (IR: 8.01 ± 1.44, *n* = 7, IR + RTX: 3.70 ± 0.81, *n* = 7; IR vs. IR + RTX, *p* = 0.037) and the number of VT episodes (IR: 12.00 ± 3.29, *n* = 7, IR + RTX: 0.57 ± 0.3, *n* = 7; IR vs. IR + RTX, *p* = 0.002) were significantly reduced in the IR + RTX group compared to the IR group (Figure 3). After 1 h of ischemia, VF occurred in five of seven animals in the IR group and two of seven animals in the IR + RTX group, with no significant difference between the groups (IR: 71% vs. IR + RTX: 29%, *p* = 0.286). In the sham group, no VT or VF was observed.

### 3.5. Immunohistochemistry

**Figure 4:**
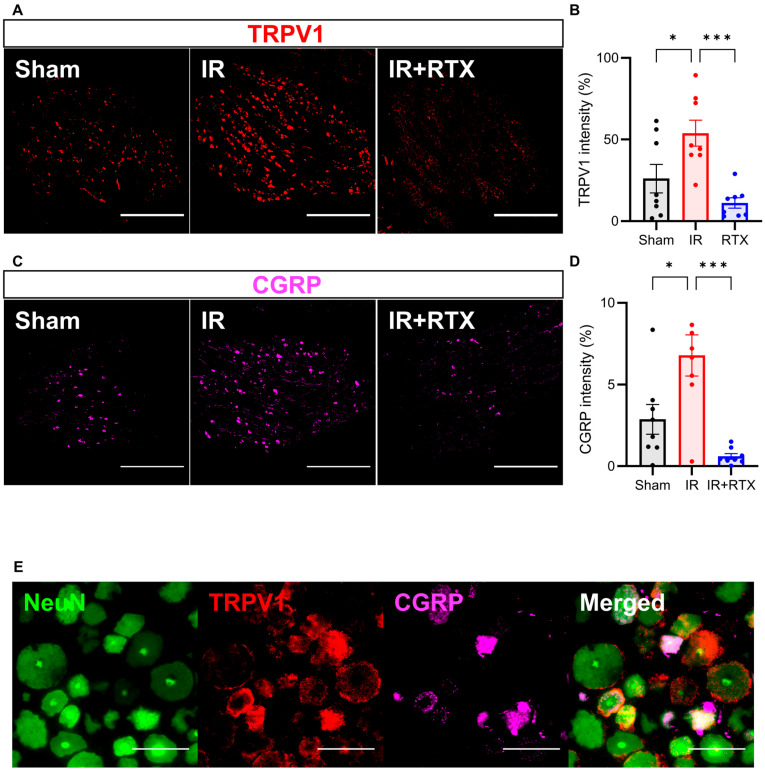
RTX reduced TRPV1 and CGRP expression in DRG. (**A**) Representative images showing TRPV1 (red) on the left DRG by immunohistochemistry. Scale bar = 1000 μm. (**B**) TRPV1 intensity in the IR + RTX group was significantly reduced compared to the IR group. (**C**) Representative images showing CGRP (pink) on the left DRG by immunohistochemistry. Scale bar = 1000 μm. (**D**) CGRP intensity in the IR + RTX group was significantly reduced compared to the IR group. (**E**) Representative images showing triple labeling of NeuN (green), a neuronal marker, TRPV1 (red), and CGRP (pink) on the left DRG in the IR group by immunohistochemistry. Scale bar = 100 μm. *n* = 8 DRGs from 3 animals per group; data are shown as mean ± SEM. * *p* < 0.05, *** *p* < 0.001 by one-way ANOVA test, followed by post hoc multiple comparisons test. TRPV1 = transient receptor potential vanilloid 1; CGRP = calcitonin gene-related peptide; DRG = dorsal root ganglion.

To confirm whether the RTX abolished TRPV1-positive neurons, we performed TRPV1 IHC of the left DRGs (T2–T4) (Figure 4A). The mean intensity of TRPV1 significantly increased as a result of the IR compared to the sham cohort. Comparatively, the treatment with RTX in the IR animals significantly decreased the intensity of TRPV1 relative to the IR animals that received no treatments (sham: 26% ± 9%, *n* = 8 DRGs, IR: 54% ± 8%, *n* = 8 DRGs, IR + RTX: 11% ± 3%, *n* = 8 DRGs; IR vs. IR + RTX, *p* < 0.001. sham vs. IR, *p* = 0.029) (Figure 4B). Since the RTX was only applied to the left DRGs, within the IR + RTX cohort, the mean intensity of TRPV1 was significantly higher in the right DRGs compared to the left (Left: 11% ± 3%, *n* = 8 DRGs vs. Right: 29% ± 8%, *n* = 7 DRGs, *p* = 0.047).

To investigate whether RTX induced CGRP expression in nociceptive neurons, we performed IHC on the T2-T4 DRGs (Figure 4C). Similar to TRPV1, the mean intensity of the CGRP significantly increased with the onset of IR compared to the sham group, and the administration of RTX in the IR animals abolished this effect (Figure 4D). (sham: 2.9% ± 0.9%, *n* = 8 DRGs, IR: 6.8% ± 1.3%, *n* = 8, IR + RTX: 0.6% ± 0.2%, *n* = 8 DRGs; IR vs. IR + RTX, *p* < 0.001. sham vs. IR, *p* = 0.015) (Figure 4). The right DRGs (that had no RTX application) displayed a significantly higher intensity of CGRP expression compared to left DRGs within the IR + RTX cohort (left: 0.6% ± 0.2%, *n* = 8 DRGs vs. right: 2.0% ± 0.4%, *n* = 9 DRGs, *p* = 0.011).

**Figure 5:**
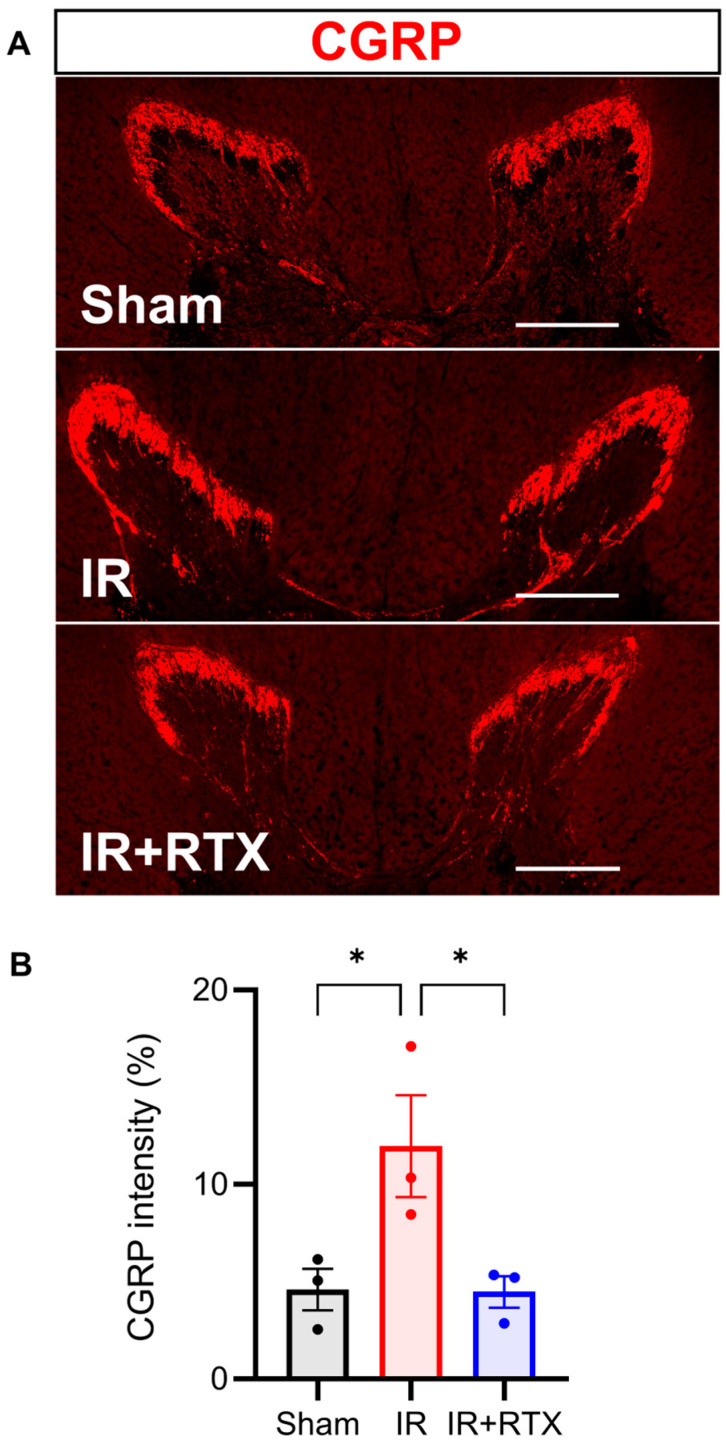
RTX attenuated CGRP expression in spinal cord. (**A**) Representative images showing CGRP (red) in thoracic 3 spinal cord by immunohistochemistry. Scale bar = 500 μm. (**B**) CGRP intensity in the IR + RTX group was significantly reduced compared to the IR group. *n* = 3 animals per group; data are shown as mean ± SEM. * *p* < 0.05 by one-way ANOVA test, followed by post hoc multiple comparisons test. CGRP = calcitonin gene-related peptide.

In addition to assessing the CGRP expression in the DRG, we also investigated spinal CGRP expression, as increased CGRP expression has been shown to increase in pathologic states in both the DRG and spinal cord [25]. In the T3 spinal cord, we found that CGRP significantly increased in the IR compared to the sham cohorts. Further, RTX in the IR animals decreased the spinal CGRP intensity expression back to baseline levels. (sham: 4.6% ± 1.1%, *n* = 3, IR: 12.0% ± 2.6%, *n* = 3, IR + RTX: 4.5% ± 0.8%, *n* = 3; IR vs. IR + RTX, *p* = 0.047. sham vs. IR, *p* = 0.049) (Figure 5).

## 4. Discussion

During myocardial ischemia, the activation of the afferent neurons leads to excessive sympathetic discharge and fatal ventricular arrhythmias [26,27,28,29]. These cardiac afferent neurons transmit the sensory information to the thoracic spinal cord dorsal horn, which activates the sympathetic preganglionic neurons in the intermediolateral column of the spinal cord, brainstem, and higher centers [30,31,32,33]. The cardiac afferent activation can be blocked by RTX at the level of the heart [4], spinal cord [14], or DRG. In this study, we have used a translational large animal model of the myocardial ischemia and we have applied the RTX selectively to the thoracic spinal dorsal root and DRG to investigate the cardioprotective effect of DRG deafferentation.

This study revealed that the application of RTX to thoracic DRGs reduces IR-triggered ventricular arrhythmogenesis in pigs without altering the hemodynamics. Our findings are supported by the following results—the application of RTX to the thoracic DRG attenuated the sympathoexcitation, arrhythmogenicity, arrhythmia score, and number of VT episodes during IR. We elucidated the underlying mechanism of action by showing that RTX blunts myocardial IR-induced TRPV1 expression in the DRG and decreases CGRP expression in the DRG and spinal cord.

### 4.1. Thoracic DRG – RTX Application Reduces Cardiac Sympathoexcitation

To investigate the cardiac electrophysiological change, we measured the ARI, a surrogate marker for the action potential duration, and DOR, a marker for arrhythmogenicity [18,20,21,22]. Our results suggest that RTX reduces sympathoexcitation (manifested by the mitigation of the ARI shortening) and suppresses arrhythmogenicity (manifested by the reduced DOR) by blocking the afferent traffic to the spinal cord. These results are supported by previous studies in which RTX was intrathecally or epidurally administered to a rat and provided cardioprotective effects [14,15,16].

In our study, the application of RTX to the thoracic DRG did not change the hemodynamic parameters as opposed to pericardial approaches. In support of our findings, RTX administered epidurally in normotensive rats similarly did not increase their blood pressure throughout 60 days [16]. These findings suggest that the epidural application of RTX to the thoracic DRG is capable of reducing arrhythmias without causing significant hemodynamic changes.

### 4.2. Molecular Mechanism Underlying Action of RTX on Thoracic DRG

Myocardial ischemia activates sensory nerve endings within the DRG, which then synapse from primary afferent DRG neurons to the thoracic dorsal horn [26,27]. These changes within the ascending pathway, in turn, cause efferent sympathoexcitation [17,26,27]. Cardiac stress leads to increased TRPV1 levels within the DRG primary afferent cells via a cAMP-dependent signaling pathway [34] and increased channel activity during myocardial ischemia. In this study, we hypothesized that blocking TRPV1 activity would lead to an improvement in the symptoms of myocardial ischemia. In order to reduce TRPV1 channel activity, we utilized RTX, which functions as a more potent analog of capsaicin that selectively affects primary neurons [9], desensitizing TRPV1 and reducing TRPV1 receptor-immunoreactivity by 35.5% [35]. When we applied RTX treatment directly to the DRG in our pig model of IR, we found a significant decrease in the intensity of TRPV1 expression in the primary afferents, which was correlated with decreased symptoms of cardiac ischemia.

The activation of TRPV1 further results in a calcium-dependent mobilization and the release of large dense-core vesicles containing CGRP [36]. This release of CGRP has been implicated in vasodilation and is reported to mediate cardioprotective effects during episodes of cardiac ischemia [36]. However, the administration of CGRP alone after cardiac ischemia did not affect the infarct size or atrial pressure in pigs [37]. Yet, the deletion of CGRP gene expression caused a decreased potentiation of the TRPV1 channel in the context of algesic sensitization [38]. These previous results indicate that the activation of the TRPV1 channels and the release of CGRP are highly correlated [39] and display significant (45%) colocalization within peptidergic DRG neurons [40]. Based on the dependent interactions previously observed between TRPV1 and CGRP, we accurately predicted that CGRP intensity would also significantly increase as a result of IR and decrease as a result of RTX application.

Our results demonstrate that RTX drives both decreases in CGRP and TRPV1 intensity while suppressing IR symptoms. We propose that this mechanism is mediated by the cessation of TRPV1 activity, which prevents the release of CGRP. Our future work will utilize the pharmacological antagonism of both TRPV1 and CGRP to confirm that TRPV1 and CGRP are necessary for the therapeutic effects of RTX during cardiac ischemia.

### 4.3. Antiarrhythmic Effect of Thoracic DRG – RTX and Clinical Implications

IR-induced arrhythmias can be triggered by excessive sympathoexcitation caused by the hyperactivation of sensory neurons [26,27,28,29], and blocking the afferent signaling during IR using various methods provides an antiarrhythmic effect [18]. In this study, we showed that a selective RTX administration suppressed arrhythmia during an IR injury. The arrhythmia score and the number of VTs were significantly reduced in the IR + RTX group compared to the IR group. An interesting finding was that the unilateral application of RTX to the thoracic DRG suppressed arrhythmogenicity. This finding is supported by the evidence that unilateral (left T2) DRG stimulation neuromodulation therapy suppressed arrhythmogenicity [18].

This new targeted approach in a preclinical model demonstrates a potentially new neuromodulation therapy for reducing sympathetic excitation and arrhythmias. The pig heart is structurally very close to the human heart and has been the subject of extensive electrophysiological research; thus, findings in this model may translate into clinical practice. The electrodes for spinal cord stimulation are routinely inserted into the epidural space in humans [41]. The lumbar epidural application of RTX for the treatment of cancer pain is in a phase II clinical trial (ClinicalTrial.gov, NCT05067257). These indicate that the results of this selective application of RTX to the thoracic DRG may be translatable for clinical use, although further safety and clinical studies are needed.

#### Study Limitations

In the present study, while the RTX was administered epidurally to the T2–T4 DRGs with a catheter, a surgical approach was performed to facilitate the access, insertion, and precise localization of the catheters in the thoracic DRGs. In patients, this approach is routinely percutaneously performed, and it is desirable to administer RTX with a less invasive approach in future studies to validate its therapeutic effect. Another consideration related to the present study design was the preemptive use and benefit of RTX during myocardial ischemia. Further studies may investigate the antiarrhythmic potential of RTX application after myocardial ischemia in subjects with refractory and repetitive chronic ischemic heart disease. Prior reports suggest that pericardial RTX had antiarrhythmic effects in a porcine model of chronic heart disease [4,42].

## Figures and Tables

**Table 1:** Hemodynamic responses to RTX.

	Baseline	5 min after RTX	30 min after RTX
HR (beats/min)	86 ± 4	86 ± 3	84 ± 3
SBP (mmHg)	129 ± 5	132 ± 5	132 ± 5
DBP (mmHg)	82 ± 4	85 ± 3	85 ± 4

Values are expressed as mean ± SEM. *n* = 7 in the IR + RTX group. HR = heart rate; SBP = systolic blood pressure; DBP = diastolic blood pressure.

**Table 2:** Hemodynamic changes to myocardial ischemia.

	Sham		IR		IR + RTX	
	Baseline	Sham30	Baseline	LAD30	Baseline	LAD30
HR (beats/min)	85 ± 4	82 ± 3	87 ± 6	88 ± 4	90 ± 5	96 ± 7
MBP (mmHg)	101 ± 8	99 ± 8	112 ± 9	104 ± 14	103 ± 5	103 ± 5
LVESP (mmHg)	110 ± 8	102 ± 9	117 ± 8	111 ± 9 *	114 ± 8	110 ± 7
dP/dt_max_ (mmHg/s)	1776 ± 79	1778 ± 101	2117 ± 186	1804 ± 167 *	1826 ± 127	1677 ± 75
dP/dt_min_ (mmHg/s)	−2751 ± 311	−2538 ± 297	−2185 ± 465	−1744 ± 258	−3499 ± 794	−3302 ± 772

Values are expressed as mean ± SEM. One-way ANOVA test was performed to compare differences in baseline measurements among the three groups. In each group, a paired Student’s *t*-test and Wilcoxon matched-pairs signed rank tests were performed to compare baseline to 30 min control recording (sham30) or 30 min after the onset of ischemia (LAD30). * *p* < 0.05. HR = heart rate; MBP = mean blood pressure; LVESP = left ventricular end-systolic pressure; dP/dt_max_ and dP/dt_min_ = maximum and minimum rate of rise in left ventricular pressure.

## Data Availability

Raw data were generated at University of Pittsburgh. Derived data supporting the findings of this study are available from the corresponding author A.M. on request.

## Abbreviations

DRG	dorsal root ganglion
RTX	resiniferatoxin
TRPV1	transient receptor potential vanilloid 1
CGRP	calcitonin gene-related peptide
IR	ischemia/reperfusion
T1	thoracic spinal nerve 1
LAD	left anterior descending coronary artery
ARI	activation recovery interval
APD	action potential duration
DOR	dispersion of repolarization
PVCs	premature ventricular contractions
VT	non-sustained ventricular tachycardias
VF	ventricular fibrillation
